# The Association of Pain and the Use of Paid Care in Older Adults with Dementia

**DOI:** 10.1093/geroni/igaf122.1842

**Published:** 2025-12-31

**Authors:** Katherine Miller, Emerald Jenkins, Tamar Rodney

**Affiliations:** Johns Hopkins Bloomberg School of Public Health, Baltimore, Maryland, United States; Johns Hopkins, University, Baltimore, Maryland, United States; Johns Hopkins, University, Baltimore, Maryland, United States

## Abstract

Chronic pain and dementia are major public health issues and risk factors for multiple comorbidities and all-cause mortality. Over half of persons living with dementia (PLWD) experience daily pain, yet pain among PLWD is often inadequately recognized and treated, which can lead to adverse health outcomes. Paid carers have been shown to prevent or mitigate behavioral and psychological symptoms, identify health status changes, and implement non-pharmacological interventions. Yet, few studies examine the intersection of pain and dementia. We use the National Health and Aging Trends study (2017-2019) to identify PLWD to examine the association of chronic pain (having bothersome or activity-limiting pain) with receipt of any paid care and intensity of paid care receipt using logistic and linear regressions, respectively, adjusting for sociodemographic and clinical characteristics (e.g., age, gender, race, depression, chronic conditions). Fifty-six percent of PLWD reported any pain. Compared to PLWD without pain, PLWD with pain are more likely to be female, Hispanic, screen positive for depression, and have more chronic conditions. Nearly 60% of PLWD with pain reported activity-limiting pain. Among PLWD, after adjusting for covariates, having pain is associated with a 5 percentage-point increase in the probability of having any paid helper (p < 0.05) but not with hours of care received. However, the association of activity-limiting pain specifically is significantly associated with an increase in the number of helpers and hours of both paid and unpaid care. These findings can help inform future interventions targeting paid and unpaid caregivers to help PLWD successfully managed pain.

